# Analysis of Rutting Formation Mechanisms and Influencing Factors in Asphalt Pavements Under Slow-Moving Heavy Loads

**DOI:** 10.3390/ma18174153

**Published:** 2025-09-04

**Authors:** Pu Li, Jiahao Fu, Linhao Sun, Jinchao Yue, Quansheng Zang

**Affiliations:** 1 School of Water Conservancy and Transportation, Zhengzhou University, Zhengzhou 450001, China; 15993684432@163.com (P.L.); fujh38542426@163.com (J.F.); yuejc@zzu.edu.cn (J.Y.); 2 Zhengzhou Public Uility Investment and Development Group Co., Zhengzhou 450001, China; 18037775771@163.com

**Keywords:** heavy-duty traffic, asphalt pavement, rutting distress, numerical simulation, thermo-mechanical coupling

## Abstract

Increasing the frequency and duration of extreme heat events significantly compromises asphalt pavement performance, particularly in critical urban infrastructure such as heavily trafficked pavements, BRT lanes, and intersections subjected to slow-moving heavy traffic under extreme temperatures. This study systematically investigates rutting formation mechanisms through integrated theoretical and numerical approaches, addressing significant knowledge gaps regarding rutting evolution under coupled extreme-temperature (70 °C), heavy-load (100 kN–225 kN), and braking conditions (1 m/s^2^–7 m/s^2^). A three-dimensional thermo-mechanical finite element model integrating solar radiation heat transfer with the Bailey–Norton creep law was developed to quantify synergistic effects of axle loads, travel speeds, and braking accelerations. Results demonstrate that when the pavement surface temperature rises from 34 °C to 70 °C, the rutting depth is increased by 4.83 times. When the axle load is increased from 100 kN to 225 kN, the rutting of conventional asphalt pavements under 70 °C is increased by 56.4%. Rutting is exacerbated by braking acceleration; due to prolonged loading duration under low acceleration, the rutting depth is increased by 30–40% compared with that under emergency braking. These findings establish theoretical foundations for optimizing pavement design and material selection in slow-moving heavy-load environments, delivering significant engineering value for transportation infrastructure.

## 1. Introduction

Temperature, as a critical factor influencing pavement material performance, remains a persistent research focus in highway engineering. Special sections such as urban heavy-traffic asphalt pavements, Bus Rapid Transit (BRT) lanes, and intersections face compounded challenges from high temperatures, heavy loads, and frequent braking, where rutting distress emerges as the primary constraint on pavement durability. Current research exhibits three fundamental limitations: (1) systematic understanding of rutting evolution under coupled extreme-temperature (70 °C), heavy-load, and braking conditions remains deficient [1,2,3]; and (2) conventional numerical models inadequately simulate braking-induced load redistribution and prolonged loading duration, failing to capture rutting mechanisms in ‘low-speed–heavy-load–braking’ scenarios [4,5,6]. Consequently, investigating rutting mechanisms under slow-moving heavy loads holds significant theoretical and practical value for enhancing service life in critical sections. This study integrates theoretical analysis and numerical simulation to elucidate synergistic mechanisms of thermal–load–braking interactions, providing theoretical foundations for design and material optimization.

Theoretical approaches to asphalt pavement rutting have evolved from simplistic constitutive models to multiphysics coupling frameworks. Early research centered on classical creep models, with the Bailey–Norton formulation extensively employed due to its parametric simplicity in characterizing temperature–stress–time-coupled creep behavior, though it neglects dynamic microstructural changes [7,8,9]. Zhu et al. (2013) developed a triaxial compression-based nonlinear viscoelastic model quantifying multiaxial stress effects on creep strain [10]. Luo et al. (2020) advanced this through a fractional-order Burgers damage creep model enabling precise real-time strain prediction under moving loads, marking the transition from linear to nonlinear theoretical frameworks [11]. Nevertheless, existing models inadequately address tripartite coupling (high-temperature–heavy-load–braking) and exhibit limited parameter alignment with actual service conditions [12,13].

Experimental methodologies have progressed from single-index to multidimensional evaluation systems. Traditional rutting tests prioritize dynamic stability (DS) as the core metric, yet demonstrate insufficient sensitivity for high-modulus mixtures with experimental errors reaching 20% under identical conditions [14,15,16,17]. To address this, Xiao et al. (2024) proposed a rutting life test using 1–1.5 cm deformation thresholds, significantly enhancing evaluation accuracy for high-modulus materials through loading-cycle quantification [3]. Li et al. 2024; Alawneh et al. 2023 established performance degradation-service life correlations via accelerated laboratory aging and full-scale field testing [18,19]. Pan et al. (2023) quantified braking acceleration effects on load redistribution through vehicle braking tests, providing empirical calibration data [20]. However, experimental replication of synergistic high-temperature–heavy-load–braking scenarios remains challenging.

With the advancement of dynamic theory, vehicle–pavement interaction models were developed to analyze rutting responses under varying temperatures, as pioneered by Jan Vrabel et al. (2017) [21]. Early static models primarily focused on vertical loading effects, with Wang et al. (2020) revealing the inhibitory function of aggregate skeletal structures against shear deformation through micro-finite element simulation [22]. Recently, thermo-mechanical coupling simulations have emerged as a research focus: Zhang et al. (2024) quantified regional climatic impacts on thermal fields and rutting by integrating solar radiation models with finite element analysis [1], while Liu et al. (2022) established the first dynamic correlation between shear stress distribution and temperature fields via 3D thermo-mechanical modeling [23]. Nevertheless, numerical simulations confront persistent challenges: insufficient accuracy in simulating load redistribution coefficients and horizontal force application during braking conditions, coupled with inadequate experimental calibration of creep parameters (e.g., A, n, and m values in the Bailey–Norton model) for composite-modified asphalts, resulting in limited engineering applicability [24,25].

Theoretical, experimental, and numerical approaches exhibit complementary limitations: theoretical simplifications overlook synergistic factor interactions; experimental constraints hinder extreme-scenario replication; numerical accuracy depends on parameter precision while balancing computational efficiency. The aim of the work is to address the unclear mechanism of rutting formation in asphalt pavements under the coupled effects of three factors—high temperature, slow-moving heavy load, and braking—through a combination of theoretical analysis, numerical simulation, and experimental verification. The specific innovations of this study include (1) the establishment of a three-dimensional thermo-mechanical finite element model integrating solar radiation heat transfer and the Bailey–Norton creep law. Combined with the load redistribution coefficient, this model quantifies the laws of rutting formation under a surface temperature of 70 °C, axle loads of 100–200 kN, and braking accelerations of 1–7 m/s^2^. (2) The high-temperature rutting resistance of instant SBS-modified asphalt, high-modulus-modified asphalt, and composite-modified asphalt under heavy-traffic conditions was systematically verified, providing experimental basis for material selection in heavy-load road sections. Experimental results indicate that under extreme high-temperature conditions, heavy loads, and frequent starting/braking, rutting increases rapidly. When the pavement surface temperature rises from 34 °C to 70 °C, the rutting depth increases by 4.83 times. Under 70 °C, when the axle load increases from 100 kN to 225 kN, the rutting of conventional asphalt pavements increases by 56.4%. Low acceleration results in a 30–40% increase in rutting depth compared to emergency braking. Experimental studies have revealed that SBS/high-modulus composite-modified asphalt mixtures exhibit excellent rutting resistance in heavy-load sections at intersections of freight corridors in high-temperature regions.

## 2. Model Establishment and Parameter Selection

### 2.1. Model Parameter Verification

Asphalt, being a viscoelastic material, exhibits significant softening susceptibility at elevated temperatures. The high-temperature stability of asphalt mixtures refers to their capacity to resist permanent deformation (primarily rutting) under repetitive traffic loading in thermally demanding environments. During summer months or on heavily trafficked road sections, pavement temperatures rise substantially, necessitating robust high-temperature stability to mitigate rutting formation. In this study, fast-dissolving SBS (SBS-T) was compounded with a high-modulus additive (LT), yielding synergistic enhancements: the high-modulus agent significantly improves rutting resistance while extending service life and enhancing durability; concurrently, the fast-dissolving SBS preserves low-temperature performance.

Currently, extreme high temperatures of asphalt pavements in most regions of China during summer typically fall within the range of 55–70 °C. For example, pavement temperatures at noon in South China during summer usually range from 60–65 °C, while extreme temperatures in the Yangtze River Basin can reach 70 °C. In the Standard Test Methods for Asphalt and Asphalt Mixtures in Highway Engineering (JTG E20-2011) [26], the standard temperature for rutting tests is set at 60 °C, which is widely adopted as the benchmark for evaluating the high-temperature stability of asphalt mixtures across the industry. As an extreme condition exceeding the standard temperature, 70 °C is used to supplementally assess material performance under more severe environmental conditions. Therefore, the selection of 60 °C and 70 °C in this study encompasses over 95% of summer extreme high-temperature scenarios in the target region, offering greater practical application value.

Given that rutting evolves through thermo-mechanical coupling (environmental temperature and dynamic loading), standard laboratory rutting tests were employed to validate finite element simulation rationality. Since rutting primarily develops within surface and intermediate layers, two gradation types were evaluated as follows: 6%-LT-modified AC-20 (it is indicated that 6% mass fraction of high-modulus agent is added as a modifier in the matrix asphalt), and 4%-SBS-T + 6%-LT-modified AC-20 (it is indicated that 4% mass fraction of SBS and 6% mass fraction of high-modulus agent are added to the matrix asphalt as modifiers). Specimens were fabricated according to standard protocols, with rutting failure defined at 10 mm deformation depth. AC-13 refers to fine-graded asphalt concrete, which is used in the surface layer of high-grade highways and the wearing course of urban arterial roads, providing skid resistance and flatness. AC-20 refers to medium-graded asphalt concrete, applied in the intermediate layer of high-grade highways and the base course of heavy-traffic roads, where it bears large loads and transmits stresses. AC-25 refers to coarse-graded asphalt concrete, utilized in the lower layer of high-grade highways, as well as the base course or subbase course of heavy-traffic roads. It primarily undertakes the functions of load transfer and structural support. High-temperature rutting resistance was assessed using a rutting tester under controlled conditions detailed in Table 1. The rut test results of various modified asphalt mixtures are shown in Table 2.

**Figure 1:**
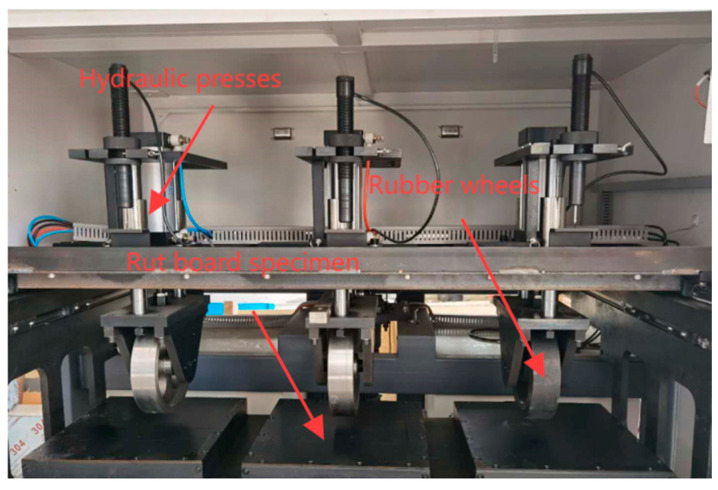
Internal device of wheel track tester.

This experiment adopted a newly developed testing device, jointly developed by Guolu Hi-Tech (Beijing, China) Engineering Technology Research Institute Co., Ltd. and Beijing Aviation Instrument Equipment Co., Ltd. (Beijing, China), with its internal structure shown in the Figure 1. This rutting testing machine is capable of independently adjusting the test temperature, tire pressure, and wheel rolling speed. The single-beam structural design is intended to ensure the stable transmission of wheel load, avoiding the problem of uneven load distribution that may occur in multi-beam structures, thereby improving the stiffness consistency of the testing system. Verified through multiple repeated tests, this evaluation method exhibits good data accuracy and discrimination, and can effectively reflect the actual rutting resistance performance of asphalt mixtures. This rutting testing machine complies with the relevant standards specified in Standard Test Methods for Asphalt and Asphalt Mixtures in Highway Engineering (JTG E20-2011) [26]. The specimen dimensions (300 × 300 × 50 mm), tire pressure (1.4 MPa), and test temperatures (60 °C, 70 °C) are all in line with the standard specifications, fully meeting the testing requirements.

**Figure 2:**
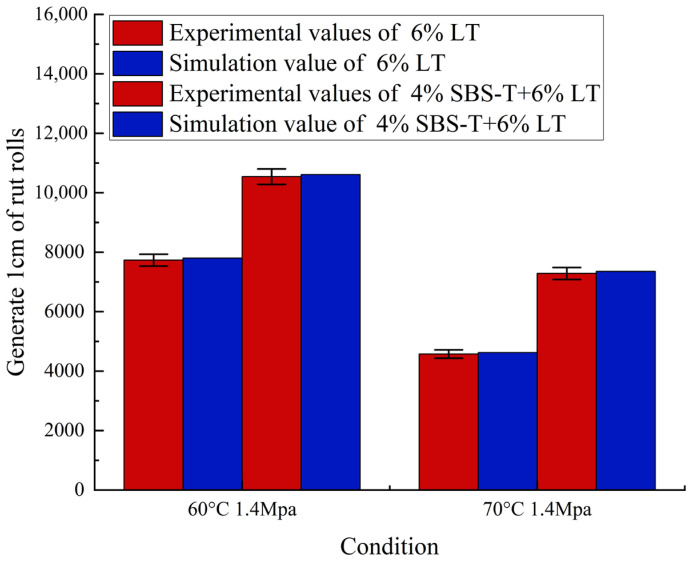
Simulation results and experimental results are verified.

Based on laboratory tests, a three-dimensional model of the single-layer plate was established for computational analysis. The test wheel traveled a distance of 230 mm ± 10 mm with a round-trip compaction speed of 30 cycles per minute and a loading duration of 2 s per cycle. Since the loading intervals were minimal relative to the total loading time, stress relaxation effects during these intervals could be neglected. Consequently, the repeated loading problem was transformed into a single-step loading scenario with total loading time applied. The load grounding dimensions had their longer sides perpendicular to the loading direction. Comparative results with experimental data are shown in Figure 2.

The comparison between indoor rutting test curves and finite element simulation results demonstrates consistent vertical displacement patterns. Post-load rutting measurements reveal 4% SBS-T + 6% LT and exhibits 4% less deformation than 6% LT, demonstrating its effectiveness in enhancing asphalt pavement’s high-temperature rutting resistance. For 6% LT rutting tests: experimental data (60 °C, 1.4 MPa/70 °C, 1.4 MPa conditions) recorded 7732/4576 cycles with simulated values of 7803/4623 cycles, showing 0.9%/1% relative error. With 4% SBS-T + 6% LT: experimental cycles (60 °C, 1.4 MPa/70 °C, 1.4 MPa conditions) recorded 10,541/7283 cycles versus simulated values of 10,613/7353 cycles, yielding 0.68%/0.96% relative error. This high correlation confirms the practicality of using finite element software for road rutting simulations.

### 2.2. Model Establishment

A three-dimensional pavement model was developed using ABAQUS (2023) finite element software, focusing on a semi-rigid asphalt pavement structure for freight transportation corridors. The computational domain was configured with dimensions of 1.92 m (length), * 1.5 m (width), and * 1.0 m (height). Full constraints were imposed at the model base (U1 = U2 = U3 = UR1 = UR2 = UR3 = 0), while lateral displacements in both horizontal directions were restricted at vertical boundaries (U1 = U3 = 0). Layer thicknesses and structural parameters were assigned according to Chinese Specification JTG D50-2017 [27].

The specific process for conducting relevant analyses in ABAQUS is as follows: First, for temperature field analysis, it is necessary to establish each structural layer of the pavement in the Part module, define material parameters in the Property module, and complete the instantiation and assembly of components in the Assembly module. Subsequently, in the Step module, steady-state and transient analysis steps are defined; in the Interaction module, thermal exchange conditions between the atmosphere and the pavement are set; in the Load module, solar radiation intensity is applied; after generating the mesh in the Mesh module, the job is submitted in the Job module for temperature field calculation. Finally, the temperature field result files can be viewed in the Visualization module. For the rutting simulation analysis under variable temperature conditions, it is carried out based on the above temperature field calculation model. It is necessary to define creep material parameters in the Property module, establish elastic analysis steps in the Step module, apply tire loads in the Load module, and finally view the rutting deformation in the Visualization module.

A load application zone was defined on the top surface, where refined meshing was implemented during discretization. The contact area of the applied load was varied according to different axle configurations. The model dimensions encompass the typical structural layers of asphalt pavements. The height of the model is sufficient to include the surface layer and intermediate layer where rutting predominantly occurs, as well as the base layer that is critical for load bearing, thereby avoiding deviations in internal force calculations caused by incomplete inclusion of structural layers. Full constraints are imposed at the bottom of the model, and horizontal displacements are restricted at the vertical boundaries; this configuration can effectively eliminate the interference of boundary effects on internal forces and deformations in the load-applied area. The length and width of the model are much larger than the tire contact area, ensuring that the load application range is entirely within the model and preventing stress concentration or deformation distortion induced by edge effects. Furthermore, in Section 2.1, the rationality of the finite element shape and mesh configuration was systematically verified through the comparison of indoor test results and numerical simulation outcomes.

**Figure 3:**
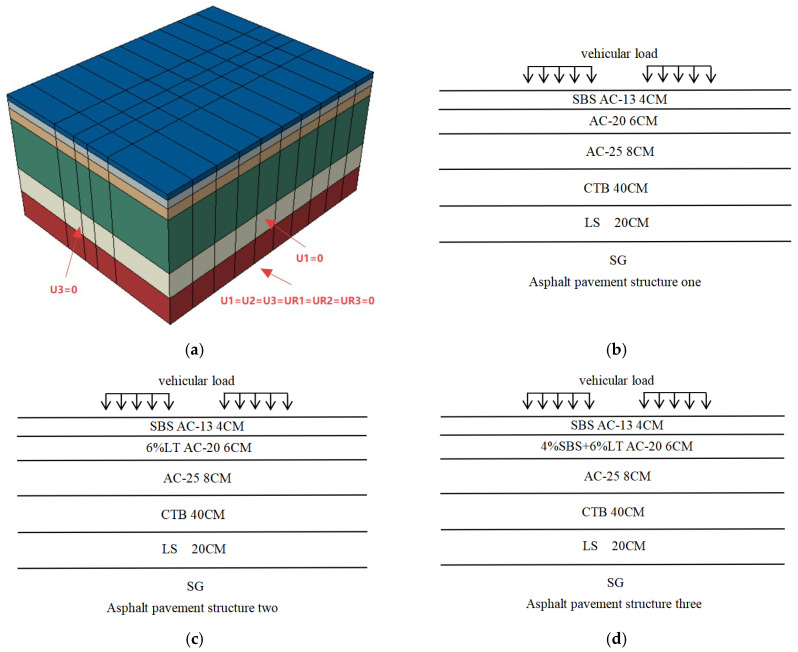
Establishment of finite element model. (**a**) Finite element model. (**b**) Asphalt pavement structure one. (**c**) Asphalt pavement structure two. (**d**) Asphalt pavement structure three.

The upper, middle, and lower layers of the asphalt pavement structure all adopt dense-graded asphalt concrete or modified dense-graded asphalt concrete (abbreviated as AC), with thicknesses of 4 cm, 6 cm, and 8 cm, respectively. The selection of these thicknesses complies with the specification in JTG D50-2017 [27] that “the total thickness of asphalt layers for extra-heavy traffic should be ≥16 cm”. The base layer is composed of cement-treated base (abbreviated as CTB) and lime soil (abbreviated as LS), with thicknesses of 40 cm and 20 cm, respectively. The subgrade is abbreviated as SG. Considering that rutting in asphalt pavements mainly occurs in the middle layer, this layer uses mixtures with enhanced high-temperature performance. The modulus of each structural layer is matched to avoid stress concentration. Specifically, the middle layer of the first pavement structure uses conventional asphalt mixture; the middle layer of the second pavement structure adopts asphalt mixture modified with high-modulus additives; and the middle layer of the third pavement structure employs composite-modified asphalt mixture (SBS + high-modulus additives). The final configuration of the finite element model is shown in Figure 3.

### 2.3. Material Parameter Selection

This study employs the Bailey–Norton creep model, a simplified simulation framework for asphalt mixture creep that describes strain evolution through independent variations of stress and time. This model essentially reflects the laws of delayed deformation of materials, as deformation accumulates with the duration of load application. When asphalt mixtures are subjected to sustained loading, the initial stage is dominated by instantaneous elastic deformation, followed by the creep stage. Under high temperatures, the viscous characteristics of the material become prominent, and deformation continues to increase over time. This process reflects the delayed deformation mechanism under the synergistic effect of stress, time, and temperature. As temperature rises or the duration of loading is prolonged, the material enters the viscous stage. At this point, n decreases significantly, and stress sensitivity declines, while m is negative, indicating that the creep rate decays over time but the total deformation continues to accumulate. The material’s creep deformation is expressed as a function of temperature T, stress σ, and time t, with the following formulation:

(1) $$\varepsilon_{cr}=Aq^{n}t^{m}$$

In this model, ε_cr_ represents the uniaxial equivalent creep strain rate, while the Parameter A is indicative of the creep characteristics of asphalt mixtures, representing the reference value of creep strain under unit stress and unit time, and its magnitude is positively correlated with temperature. When temperature rises, the bonding force of asphalt is weakened, and the value of A increases significantly. Parameter n describes the sensitivity of creep strain to stress. A larger n value indicates that the influence of stress variation on creep deformation is more significant. At low temperatures, asphalt mixtures are dominated by elasticity, with the n value being close to 1, and the relationship between stress and deformation is approximately linear; at high temperatures, the n value decreases, leading to reduced stress sensitivity. Parameter m reflects the development law of creep strain over time, is usually a negative value, and indicates the attenuation characteristic of creep rate. A smaller m value suggests that the growth rate of creep strain slows down more significantly as time elapses. The creep characteristics of this model are mainly reflected by the time term tm. The model incorporates the coupling effects of temperature, stress, and time through parameter A and stress order n, thereby accurately describing the characteristics that creep is intensified after asphalt materials soften at high temperatures and deformation is accelerated due to stress concentration under heavy loads.

For SBS-modified asphalt concrete, the values of A, m, and n were referenced from paper [28]. Since rutting predominantly develops within intermediate pavement layers, structural optimization was implemented using 6%-LT-modified AC-20 and 4%-SBS + 6%-LT-modified AC-20 mixtures. Material parameters were subsequently calibrated against laboratory rutting tests to ensure computational accuracy of the finite element model. Based on the above empirical formula and according to the engineering empirical method and the value of Poisson’s ratio and elastic modulus parameters in relevant papers [29], this paper takes the elastic parameters of asphalt mixture at different temperatures as shown in Table 3 and Table 4.

To comprehensively characterize the thermal regime within asphalt pavement structures, heat transfer analysis was conducted using ABAQUS finite element software. A pavement structural model was established within the computational environment, where thermal conductivity coefficients were assigned to individual layers. Through extensive literature review, thermophysical parameter ranges were determined for diverse mixture types across structural layers. Solar radiation, ambient temperature, convective heat exchange, and effective pavement radiation were systematically incorporated. The 24 h temporal temperature distribution throughout the pavement structure was subsequently computed. The thermal simulation was configured based on recorded high-temperature conditions in an urban environment, with ambient temperature data provided in Table 5.

The thermal characteristics of asphalt pavement structure material parameters are shown in Table 6. In this table, thermal conductivity refers to the ability of a material to conduct heat, which is a physical quantity that measures the rate at which heat is transferred within the material through mechanisms such as molecular vibration. A larger value indicates stronger thermal conductivity of the material, meaning heat is more easily transferred inside it. Heat capacity is the amount of heat absorbed (or released) by a unit mass of material when its temperature increases (or decreases) by 1 °C, reflecting the material’s ability to store heat. A larger value means the material can absorb or release more heat when its temperature changes. Solar radiation absorption rate is the ratio of the solar radiation energy absorbed by a material to the total solar radiation energy incident on its surface, with a value range of 0 to 1. A value closer to 1 indicates a stronger ability of the material to absorb solar radiation. Surface emissivity, also known as emissivity, is the ratio of the energy radiated per unit time by a material’s surface to the energy radiated by the surface of an ideal black body (an object that can completely absorb and radiate all energy) at the same temperature, with a value range of 0 to 1. A larger value indicates a stronger ability of the material to release energy through thermal radiation. Stefan–Boltzmann constant: a fundamental physical constant in thermodynamics, used to describe the relationship between the radiant power of a black body and temperature. This constant is the core parameter of the Stefan–Boltzmann law, which states that the radiant power per unit area of a black body is proportional to the fourth power of its thermodynamic temperature.

### 2.4. Tire Contact Area and Loading Duration

Tire pressure plays a critical regulatory role: under high inflation pressure, area expansion is predominantly length-driven with negligible width variation; conversely, low pressure may induce simultaneous length and width increases. Under extreme loading, nonlinear contact area expansion is observed, potentially causing non-uniform tread pressure distribution and geometric irregularity. For modeling simplification, width deformation was disregarded while contact length extension was incorporated, because the actual grounding area calculated by the tire grounding pressure is not completely equal to the tire internal pressure. Therefore, the grounding pressure under the load can be calculated according to the empirical formula [30,31]:

(2) $$p=0.290p_{t}+0.0042P+0.1448$$

where p is the tire ground pressure, MPa; p_t_ is the tire inflation pressure, MPa; and P is the axle load, kN.

Frequent braking and acceleration at intersections induce significant load transfer from rear to front axles, accompanied by substantial horizontal forces. During braking, front-axle loads increase while rear-axle loads decrease compared to steady-state conditions, with load magnitude and deceleration rate being primary influencing factors. This process imposes considerable horizontal forces on pavements while redistributing axle loads. The load redistribution coefficient for fully loaded vehicles was adopted from Ma Huixu’s computational results [32].

Conventional standard axle load models become inadequate under heavy loads due to simultaneous variations in tire contact area and inflation pressure. Consequently, loading configurations must be redefined based on overload-specific contact geometry and pressure parameters. Traditional overload simulations typically maintain constant contact pressure while increasing axle load and contact area—an approach misaligned with actual practice where heavy vehicles employ high-strength tires and elevated inflation pressures.

The tire–pavement contact area during braking (A) is calculated as follows:

(3) A=LB

(4) $$L=\frac{\delta P}{n_{w}pB}$$

In this formula, B is the tire ground width, cm; L is the tire ground length, cm; and δ is the axle load distribution coefficient.

In freight corridor intersections characterized by high heavy-vehicle proportions and substantial axle loads, deceleration or idling during red phases induces critical braking scenarios. During braking maneuvers, center-of-gravity shift toward the front axle increases front-axle loading, with peak pavement distress occurring near complete stoppage. Consequently, the travel velocity was approximated by the speed at 1 m prior to standstill in simulations. The computational expression for cumulative loading duration is formulated as follows:

(5) $$T=\frac{L}{V}=\frac{\delta PN}{n_{w}pB\sqrt{2a}}$$

In the formula, V represents the speed $\sqrt{2a}$ of the vehicle 1 m before braking; a denotes the acceleration during braking, m/s^2^; T is the cumulative wheel load time in seconds; B is the tire ground width in cm; P is the axle load in kN; $n_{w}$ indicates the number of wheels per axle; N is the number of wheel load cycles; and p stands for the tire ground pressure in MPa.

According to Dong Niya’s axle load survey on an operational expressway [33], overloaded vehicles constituted nearly or exceeding 50% of traffic during initial service years, with progressive annual reductions observed as the pavement aged. By 2016, axle load distribution demonstrated 87.35% within 80–120 kN, 12.6% exceeding 120 kN, and 0.05% surpassing 200 kN. According to the previous calculation results, the relationship between acceleration, axle load, ground pressure, and tire grounding length during brake start under typical conditions is shown in Table 7.

## 3. Computation and Analysis

### 3.1. Temperature Field Analysis and Validation

**Figure 4:**
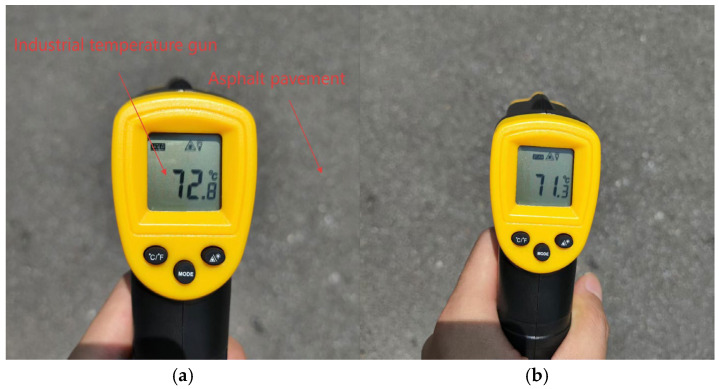
(**a**,**b**) Measured surface temperature of asphalt road.

Accelerated industrial development has intensified greenhouse effects, particularly in coastal regions where rising ambient temperatures and prolonged heatwaves are increasingly observed. These elevated temperatures detrimentally impact asphalt pavement performance, as thermal elevation degrades material mechanical properties and exacerbates rutting susceptibility. Field measurements during summer conditions recorded peak pavement temperatures of 72.8 °C locally, with averages reaching 71.3 °C. The standard deviation of the road surface temperature measurement result is ≤1 °C as documented in Figure 4a,b.

Data were systematically collected in Zhengzhou on 15 July under extreme high-temperature climatic conditions, with measurements conducted during the peak solar insolation period (12:00–14:00 h). The ambient air temperature remained constant at 41 °C throughout the testing window, with wind speeds ≤ 2 m/s and no precipitation recorded, conditions that effectively mitigated significant disturbances from atmospheric turbulence or water vapor on pavement temperature dynamics. The test segment comprised a heavy-load lane of an urban arterial roadway, characterized by a typical black surface finish with a solar radiation absorption coefficient of 0.9. The pavement exhibited no discernible wear or bleeding phenomena, ensuring homogeneous heat absorption properties across the measurement domain. The testing area was specifically selected as a straight, grade-free roadway section to eliminate confounding variables associated with slope-induced variations in solar exposure duration. The test section is the heavy-load lane of the urban main road, with an average daily traffic volume of 1200 large trucks and 8000 small buses. Temperature measurements were performed using an industrial-grade infrared thermometer (supplier: Deli) with specified technical parameters: measurement accuracy of ±2 °C, resolution of 0.1 °C, temperature measurement range of −30 °C to 380 °C, and response time of 500 ms. Measurement loci were randomly distributed within the wheel path zone of the lane, and triplicate measurements were recorded at each sampling point with arithmetic mean values calculated to minimize single-point measurement errors, adhering to rigorous experimental protocols for data reliability and precision.

Asphalt mixtures exhibit pronounced temperature sensitivity, manifesting distinct mechanical responses across thermal regimes. At low temperatures, predominantly elastic behavior with a high modulus is observed. Conversely, progressive modulus reduction occurs during visco-plastic transition under thermal elevation. The temperature field distribution was computationally established to quantify thermally induced modulus variations within pavement structures.

Based on Yan Zuoren’s asphalt pavement temperature field theory [14,34], the thermal regime was simulated by integrating three mechanisms: convective heat exchange between pavement surface and air, solar radiation absorption, and effective thermal radiation emission. Convective heat exchange was modeled using the FILM subroutine with daily maximum/minimum temperatures and average wind speed as inputs. Solar radiation was implemented via the DFLUX subroutine incorporating total solar irradiance and sunshine duration.

**Figure 5:**
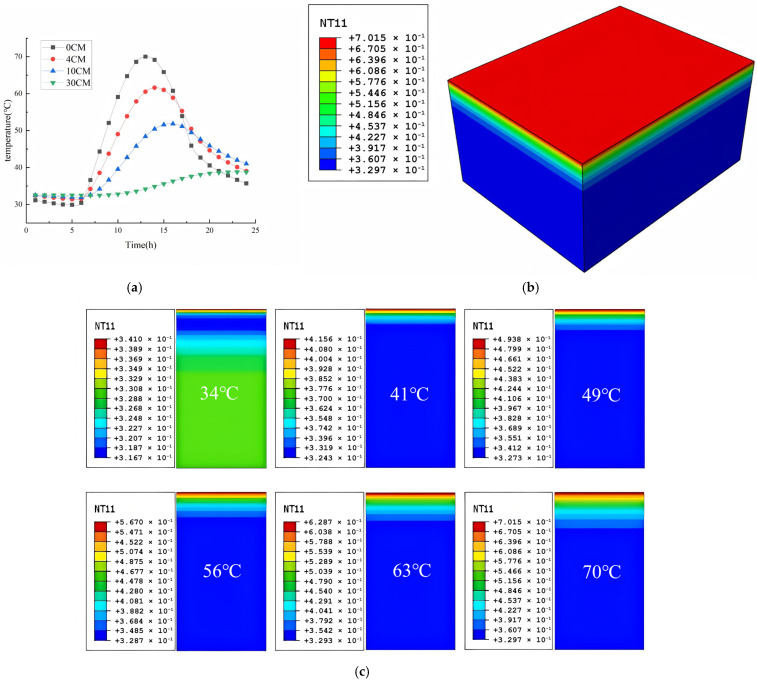
Temperature field simulation results. (**a**) Simulated calculation of temperature field variation trend of each layer of asphalt pavement in 24 h. (**b**) Calculations of the distribution value of the temperature field at 70 °C on the road surface. (**c**) Temperature field distribution under different road surface temperatures.

**Figure 6:**
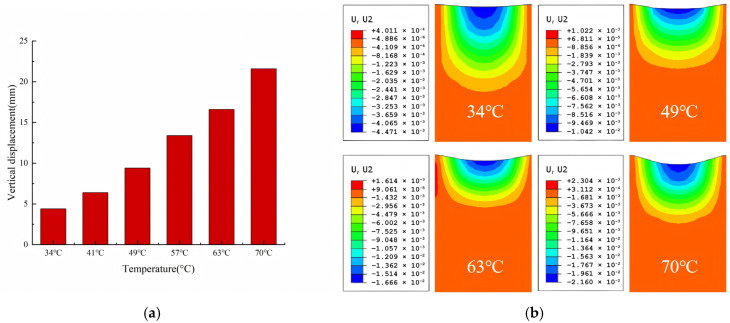
Influence of different temperatures on rutting of asphalt pavement. (**a**) Vertical rut displacement at different temperatures. (**b**) Track rolling at typical temperatures.

Figure 5a illustrates the 24 h thermal evolution within the asphalt pavement structure, revealing peak surface temperatures of 70 °C under sustained extreme conditions, with subsurface temperatures progressively attenuating to 63 °C at the upper-layer base, 55 °C in the intermediate layer, and 44–50 °C in the base course due to conductive and radiative heat dissipation. Surface temperatures (0 cm depth) exhibit pronounced diurnal fluctuations driven by direct solar radiation absorption, while deeper layers demonstrate enhanced thermal stability with minimal environmental influence. Distinct thermal hysteresis is observed across depth profiles, where subsurface temperatures lag surface variations due to finite thermal conduction rates. Computational analyses at six characteristic surface temperatures (34 °C, 41 °C, 49 °C, 57 °C, 63 °C, and 70 °C) are visualized in Figure 6b, confirming depth-dependent thermal response patterns.

### 3.2. Influence of Different Temperatures on Rutting of Asphalt Pavement

**Figure 7:**
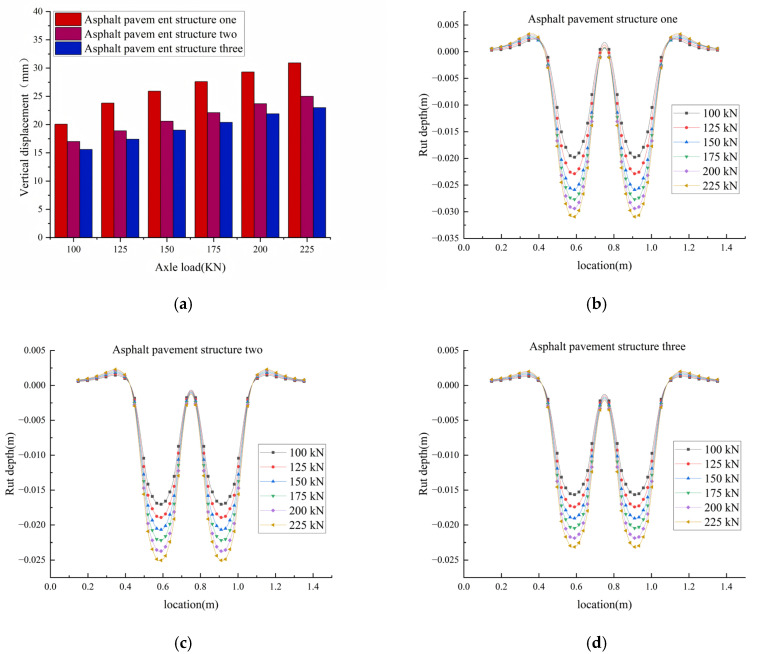
Rut calculation results. (**a**) Influence of axle load on rutting on asphalt pavement. (**b**) Asphalt pavement structure one rutting occurs. (**c**) Asphalt pavement structure two rutting occurs. (**d**) Asphalt pavement structure three rutting occurs.

Figure 6a simulates vertical rutting displacement in SBS-modified asphalt pavement under 10,000 loading cycles with 3 m/s^2^ braking acceleration at varying surface temperatures. Figure 7b presents displacement contour plots of selected computational results and the specific calculation results are given in Table 8. When temperature increased from 34 °C to 70 °C, rut depth was amplified by 4.83 times. This phenomenon is attributed to the rapid modulus degradation of asphalt mixtures during thermal elevation, inducing a progressive transition from elastic solid to viscoelastic fluid state.

The core findings of this study are consistent with those of comparable research, but the understanding of the coupling mechanism is deepened through quantitative data. Zhang (2024) [1] indicated that extreme high temperatures significantly exacerbate rutting in asphalt pavements. This study further quantifies the influence of temperature ranges—when the temperature rises from 34 °C to 70 °C, the rutting depth increases by 4.83 times (Figure 7a), clarifying the quantitative relationship between high temperatures and rutting formation and addressing the lack of a description of specific temperature interval effects in previous studies.

Based on quantitative analyses of six representative pavement surface temperatures (34 °C, 41 °C, 49 °C, 57 °C, 63 °C, and 70 °C), the impact of temperature on rutting exhibits stage-specific differences:

Medium-temperature range (34–49 °C): The rutting depth increases from approximately 4 mm to 8 mm. The elastic modulus of the asphalt mixture decreases from 1031 MPa to 810 MPa, with the material still dominated by elasticity and deformation growing gradually. Under these conditions, traffic can operate normally without significant rutting risks.

Higher-temperature range (49–63 °C): The rutting depth increases from 8 mm to 13 mm. The elastic modulus decreases to 552–400 MPa, with viscosity dominance increasing and deformation rates accelerating. Enhanced pavement inspections are required during these high-temperature periods to avoid concentrated passages of heavy-load vehicles.

Extreme high-temperature range (63–70 °C): The rutting depth surges from 13 mm to 19 mm. The modulus drops to 248 MPa, and the material approaches a visco-fluid state, making it prone to irreversible flow deformation under wheel loads. Traffic control measures should be strengthened within this temperature range to restrict the passage of heavy-load vehicles.

From a rheological perspective, thermal elevation fundamentally alters mixture behavior: Elastic components diminish while viscous constituents dominate, reducing instantaneous elastic deformation but increasing delayed viscous strain. This time-dependent response results in significantly greater cumulative permanent strain under identical loading at elevated temperatures compared to ambient conditions.

### 3.3. Influence of Axle Load on Asphalt Rutting Under High Temperature

In this chapter and subsequent chapters, three pavement structures are involved. These structures share the same materials for the surface layer, lower layer, and base layer: the surface layer is constructed with SMA-13, the lower layer with AC-25, the base layer with cement-stabilized macadam and lime soil, and the bottom-most layer is the subgrade. However, they differ in the material used for the intermediate layer: asphalt pavement structure one employs a conventional asphalt mixture for the intermediate layer; asphalt pavement structure two utilizes an asphalt mixture modified with high-modulus additives in the intermediate layer; and asphalt pavement structure three adopts a composite-modified asphalt mixture (SBS + high-modulus additives) for the intermediate layer.

Compared to temperature, overloading constitutes another significant accelerator of rutting. Given that rutting under ambient temperatures is negligible relative to high-temperature conditions, this analysis exclusively examines axle load effects on three pavement structures at 70 °C.

Figure 7 presents the simulated vertical rutting displacement magnitudes and their spatial distribution across the pavement profile for structures one, two, and three under the following conditions: a pavement surface temperature of 70 °C, varying axle loads, a constant braking acceleration of 3 m/s^2^, and 10,000 load repetitions, the specific calculation results are given in Table 9. At 70 °C, when the axle load was increased from 100 kN to 200 kN, the rutting depths of structure 1, structure 2, and structure 3 increased by 48.6%, 39.3%, and 39.7%, respectively. This indicates that increased axle weights significantly elevate the contact pressure at the tire–pavement interface, thereby subjecting asphalt mixtures to greater vertical compressive and shear stresses. From the perspective of pavement structure, increased axle weights induce higher shear stresses in the lower region of the asphalt surface layer. When these stresses exceed the shear strength of the mixture, interlayer shear slip occurs, leading to rutting formation. Additionally, if the base layer or subgrade exhibits insufficient stiffness, the load transmitted to these underlying layers may induce structural deformation, further deepening the ruts. Meanwhile, increased axle weights accelerate fatigue damage within the mixture. This damage process promotes the propagation of micro-cracks, reduces the overall stiffness of the material, and ultimately accelerates deformation rates.

As shown in the table, under a 100 kN load, the rutting amounts of structure 2 and structure 3 were reduced by 2.76 mm and 4.13 mm, respectively; under a 200 kN load, the rutting amounts were reduced by 5.68 mm and 7.52 mm, respectively. The calculation results demonstrate that the addition of SBS modifiers and high-modulus agents significantly improves material performance. Among them, the composite-modified asphalt mixture (SBS + high-modulus additive) exhibits the best high-temperature rut resistance, making it most suitable for heavy-load sections in high-temperature regions, such as key freight corridors and intersections.

### 3.4. Influence of Braking on Rutting on Asphalt Pavement

Vehicle braking and acceleration maneuvers at intersections, particularly under low-speed operation with load transfer to front axles compounded by high-temperature conditions, significantly exacerbate rutting in asphalt pavements. This section specifically examines braking acceleration effects on rut formation at 70 °C surface temperature.

**Figure 8:**
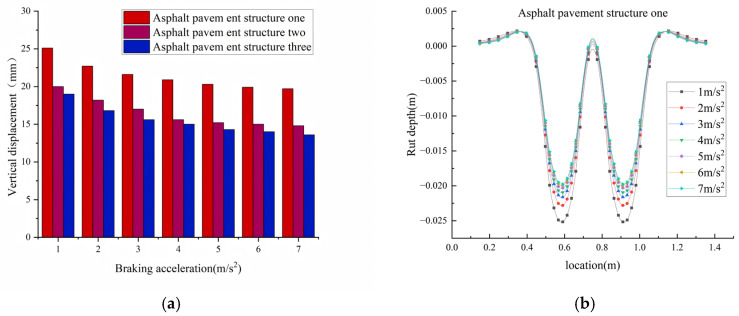

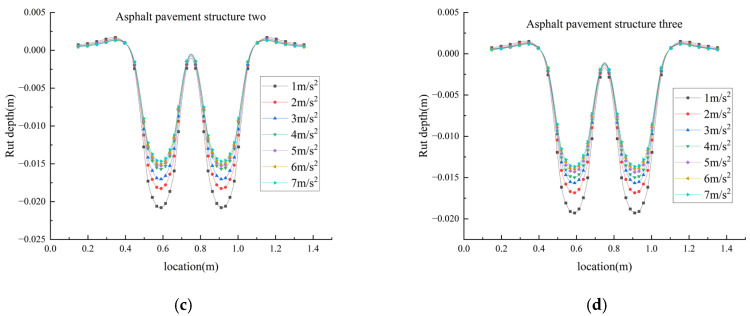
Rut calculation results. (**a**) Influence of braking acceleration on rutting on asphalt pavement. (**b**) Asphalt pavement structure one rutting occurs. (**c**) Asphalt pavement structure two rutting occurs. (**d**) Asphalt pavement structure three rutting occurs.

Figure 8 presents the simulated vertical rutting displacement distribution across the pavement profile for structures one and two under the following conditions: a pavement surface temperature of 70 °C, an axle load of 100 kN, varying levels of braking acceleration, and 10,000 load repetitions, the specific calculation results are given in Table 10. Li et al. (2015) [35] noted that braking loads exacerbate rutting but did not differentiate between acceleration intervals; this study clarifies the differences in rutting increments within the 1–7 m/s^2^ range. As shown in the table, at 70 °C, when the braking acceleration decreased from 7 m/s^2^ to 1 m/s^2^, the rutting depths of structure 1, structure 2, and structure 3 increased by 27.5%, 41.8%, and 41.1%, respectively. The results indicate that lower braking accelerations prolong the duration of sustained loading exerted by vehicles on the pavement, and such prolonged loading intensifies the creep deformation of the mixture under high-temperature conditions. As a temperature-sensitive viscoelastic material, asphalt exhibits significant viscous flow under sustained loading. This characteristic is particularly prominent in summer—high temperatures lead to a sharp decline in material modulus, significantly weakening the deformation resistance of the mixture and thereby accelerating rutting formation.

Under the braking acceleration of 1 m/s^2^, the rutting amounts generated by structure 2 and structure 3 were reduced by 4.37 mm and 5.88 mm, respectively. Under a braking acceleration of 7 m/s^2^, the rutting amounts of structure 2 and structure 3 were reduced by 5.08 mm and 6.08 mm, respectively. The calculation results demonstrate that the rutting resistance of structure 2 and structure 3 is significantly superior to that of structure 1. This improvement is attributed to the application of SBS modifiers and high-modulus additives—which not only enhance the high-temperature rutting resistance of asphalt mixtures but also increase their elastic modulus under low-frequency loading, thereby comprehensively improving the rutting resistance of the pavement system. Therefore, modified asphalt concrete incorporating SBS modifiers and high-modulus additives is more suitable for road sections with frequent starting and braking in high-temperature regions, such as intersections along key freight corridors.

## 4. Conclusions

This study systematically investigated rutting formation mechanisms and contributing factors in three distinct asphalt pavement structures under extreme high-temperature conditions and slow-moving heavy traffic, employing a three-dimensional thermo-mechanical finite element model that integrates solar radiation heat transfer with the Bailey–Norton creep law. The principal conclusions are summarized as follows:

(1) When the pavement temperature rises from 34 °C to 70 °C, the rutting depth increases by 4.83 times. Within the extreme high-temperature range of 63–70 °C, the material approaches a visco-fluid state, with enhanced fluidity of asphalt mortar and weakened aggregate bonding, which easily induces irreversible permanent deformation. When the axle load increases from 100 kN to 200 kN (100% overloading), under 70 °C, the rutting depths of the three pavement structures increase by 48.6%, 39.3%, and 39.7%, respectively. High axle loads cause the maximum shear stress zone to shift from the surface layer to the intermediate layer, exacerbating interlayer slip and aggregate rearrangement, which act as key triggers for permanent deformation. Low braking acceleration (≤3 m/s^2^) leads to more significant creep deformation of the mixture under high temperatures, with the rutting depth increasing by 30–40% compared to that under emergency braking (7 m/s^2^). This is related to the synergistic effect of reduced dynamic modulus of asphalt materials and prolonged viscous flow time.

(2) Numerical simulation studies on pavement structures indicate that asphalt mixtures modified with a composite of SBS and high-modulus additives can significantly enhance the high-temperature rutting resistance of asphalt pavements. Under conditions of 70 °C and 200 kN axle load, its rutting depth is reduced by 7.52 mm compared to conventional structures. Therefore, SBS/high-modulus composite-modified asphalt mixtures are suitable for heavy-load sections at intersections of freight corridors in high-temperature regions.

(3) This study quantifies the influence of temperature in the range of 34–70 °C on rutting formation, providing refined temperature zoning parameters for pavement design in high-temperature regions and optimizing structural thickness and material selection for heavy-load sections. Based on field-measured temperature field data and rutting prediction laws derived from finite element models, this study can support the development of an intelligent monitoring system integrating infrared temperature measurement and strain sensing, enabling real-time early warning of rutting risks for key sections such as freight corridors and BRT lanes. Additionally, this study verifies the potential of composite modification technology to enhance the performance of traditional asphalt mixtures, providing data support for the engineering application of instant SBS modifiers and high-modulus agents. This reduces resource consumption caused by frequent maintenance, aligning with the development needs of green transportation infrastructure.

(4) Several limitations exist in this study, and there remain multiple aspects in the numerical calculation of rutting in pavement structures that can be subjected to more refined and comprehensive follow-up studies. The simulation of contact behavior between tires and pavement in the current model is performed by simplifying the contact pressure to a uniform distribution. In follow-up studies, more realistic tire models accounting for tread deformation and non-uniformly distributed contact pressure may be considered, integrated with dynamic braking processes involving factors such as variable acceleration and tire slip. This would further reveal the microscopic mechanism by which transient stress fields induce and propagate rutting, thereby improving the computational accuracy of the model. The constitutive relation of plastic deformation has not been incorporated into the model during calculations. In future research, the constitutive relation of plastic deformation could be introduced to investigate the influence of plastic deformation in pavement structures on rutting.

## Figures and Tables

**Table 1:** Test condition parameters of wheel track test.

Test Parameters	Test Condition 1	Test Condition 2
Test specimen Specifications	300 mm × 300 mm × 50 mm	300 mm × 300 mm × 50 mm
Test piece temperature	60 °C	70 °C
Tire size	Steel, wheel pressure 1.4 MPa	Steel, wheel pressure 1.4 MPa
Rolling distance	230 mm × 10 mm	230 mm × 10 mm
Rolling speed	30 times per minute	30 times per minute

**Table 2:** Results of wheel track test.

Type of Mix	Generate 1 cm Wheel Track Action Times		Technical Requirement	Experimental Method
	60 °C, 1.4 MPa	70 °C, 1.4 MPa		
6%LT	7732	4576	—	T0719
4%SBS-T + 6%LT	10,541	7283		

**Table 3:** Model material parameters.

Material Type	Temperature (°C)	Modulus of Resilience (E/MPa)	Poisson’s Ratio	A	n	m
AC-13	20	1452	0.2	6.54 × 10^−11^	0.947	−0.591
	30	1031	0.25	3.33 × 10^−9^	0.872	−0.584
	40	810	0.3	1.44 × 10^−8^	0.797	−0.575
	50	552	0.35	1.39 × 10^−6^	0.418	−0.523
	60	400	0.4	1.46 × 10^−5^	0.336	−0.502
	70	248	0.45	2.78 × 10^−5^	0.258	−0.479
AC-20	20	1200	0.2	5.32 × 10^−11^	0.939	−0.589
	30	1000	0.25	4.09 × 10^−9^	0.843	−0.581
	40	900	0.3	7.26 × 10^−8^	0.801	−0.569
	50	800	0.35	5.29 × 10^−6^	0.513	−0.527
	60	600	0.4	6.26 × 10^−5^	0.365	−0.433
	70	400	0.45	1.20 × 10^−4^	0.217	−0.339
AC-25	20	1289	0.2	6.49 × 10^−11^	0.915	−0.572
	30	1100	0.25	5.67 × 10^−9^	0.819	−0.562
	40	910	0.3	6.45 × 10^−8^	0.777	−0.54
	50	852	0.35	1.27 × 10^−6^	0.489	−0.502
	60	620	0.4	4.08 × 10^−5^	0.341	−0.454
	70	388	0.45	8.03 × 10^−5^	0.193	−0.386
6% LT AC-20	60	504	0.4	1.01 × 10^−5^	0.343	−0.504
	70	341	0.45	2.78 × 10^−5^	0.277	−0.487
4% SBS + 6% LT AC-20	60	1093	0.4	6.91 × 10^−6^	0.325	−0.473
	70	740	0.45	3.1 × 10^−5^	0.217	−0.429

**Table 4:** Model material parameters.

Material	Modulus of Resilience E/MPa	Poisson’s Ratio
CTB	1200	0.2
LS	300	0.3
SG	45	0.4

**Table 5:** Actual temperature was measured for 24 h.

Time (h)	Temperature (°C)	Time (h)	Temperature (°C)	Time (h)	Temperature (°C)
1	31	9	30	17	39
2	30	10	32	18	37
3	30	11	33	19	36
4	29	12	36	20	35
5	28	13	40	21	35
6	27	14	41	22	34
7	25	15	41	23	33
8	27	16	41	24	32

**Table 6:** Thermodynamic material parameters.

Parameter	Asphalt Layer	CTB	LS	SG
Thermal conductivity	4680	5616	5148	5616
Density	2300	2200	2100	1800
Heating capacity	924.9	911.7	942.9	1040
Solar radiation absorption rate	0.9			
Surface emissivity	0.81			
Absolute zero	−273			
Stefan–Boltzmann	2.041 × 10^−4^			

**Table 7:** The relationship between acceleration, axle load, ground pressure, and tire grounding length.

Accelerated Speed	1	2	3	4	5	6	7
Shaft load Distribution coefficient	0.032	0.065	0.097	0.13	0.162	0.195	0.227
The axle load is standard axle load 100 kN							
Forward shift weight P (kN)	103.2	106.5	109.7	113	116.2	119.5	122.7
Ground pressure (MPa)	0.78	0.8	0.81	0.82	0.84	0.85	0.86
L (m)	0.178	0.18	0.182	0.185	0.187	0.189	0.191
The axle load is overloaded by 50% and the axle load is 150 kN							
Forward shift weight P (kN)	154.8	159.8	164.6	169.5	174.3	179.3	184.1
Ground pressure (MPa)	1	1.02	1.04	1.06	1.08	1.1	1.12
L (m)	0.208	0.21	0.213	0.215	0.217	0.219	0.221
The axle load is overloaded by 100% and the axle load is 200 kN							
Forward shift weight P (kN)	206.4	213	219.4	226	232.4	239	245.4
Ground pressure (MPa)	1.21	1.24	1.27	1.3	1.32	1.35	1.38
L (m)	0.228	0.23	0.232	0.234	0.236	0.238	0.239

**Table 8:** Vertical displacement under different temperature conditions (m).

Temperature	34 °C	41 °C	49 °C	57 °C	63 °C	70 °C
Structure 1	0.00447	0.00643	0.01042	0.01341	0.01666	0.02160

**Table 9:** Calculation results of vertical displacement under different axial loads (m).

Axle Load	100 kN	125 kN	150 kN	175 kN	200 kN	225 kN
Structure 1	−0.01978	−0.02287	−0.02587	−0.02768	−0.02939	−0.03094
Structure 2	−0.01702	−0.01892	−0.02067	−0.02219	−0.02371	−0.02504
Structure 3	−0.01565	−0.01741	−0.01904	−0.02046	−0.02187	−0.02311

**Table 10:** Vertical displacement under different braking acceleration conditions (m).

Brake Acceleration	1 m/s^2^	2 m/s^2^	3 m/s^2^	4 m/s^2^	5 m/s^2^	6 m/s^2^	7 m/s^2^
Structure 1	−0.02516	−0.02278	−0.02160	−0.02094	−0.0203	−0.01996	−0.01974
Structure 2	−0.02079	−0.01827	−0.01702	−0.01570	−0.01527	−0.01503	−0.01466
Structure 3	−0.01928	−0.01684	−0.01565	−0.01498	−0.01430	−0.01397	−0.01366

## Data Availability

The original contributions presented in this study are included in the article. Further inquiries can be directed to the corresponding author.
